# A stratified treatment algorithm in psychiatry: a program on stratified pharmacogenomics in severe mental illness (Psych-STRATA): concept, objectives and methodologies of a multidisciplinary project funded by Horizon Europe

**DOI:** 10.1007/s00406-024-01944-3

**Published:** 2024-12-27

**Authors:** B. T. Baune, S. E. Fromme, M. Aberg, M. Adli, A. Afantitis, I. Akkouh, O. A. Andreassen, C. Angulo, S. Barlati, C. Brasso, P. Bucci, M. Budde, P. Buspavanich, V. Cavone, K. Demyttenaere, C. M. Diaz-Caneja, M. Dierssen, S. Djurovic, M. Driessen, U. W. Ebner-Priemer, J. Engelmann, S. Englisch, C. Fabbri, P. Fossati, H. Fröhlich, S. Gasser, N. Gottlieb, E. Heirman, A. Hofer, O. Howes, L. Ilzarbe, H. Jeung-Maarse, L. V. Kessing, T. D. Kockler, M. Landén, L. Levi, K. Lieb, N. Lorenzon, J. Luykx, M. Manchia, M. Martinez de Lagran, A. Minelli, C. Moreno, A. Mucci, B. Müller-Myhsok, P. Nilsson, C. Okhuijsen-Pfeifer, K. D. Papavasileiou, S. Papiol, A. F. Pardinas, P. Paribello, C. Pisanu, M. -C. Potier, A. Reif, R. Ricken, S. Ripke, P. Rocca, D. Scherrer, C. Schiweck, K. O. Schubert, T. G. Schulze, A. Serretti, A. Squassina, C. Stephan, A. Tsoumanis, E. Van der Eycken, E. Vieta, A. Vita, J. T. R. Walters, D. Weichert, M. Weiser, I. R. Willcocks, I. Winter-van Rossum, A. H. Young, M. J. Ziller

**Affiliations:** 1 Department of Psychiatry, University of Muenster, Muenster, Germany; 2 Department of Medical Science, Clinical Chemistry and SciLifeLab Affinity Proteomics, Uppsala University, Uppsala, Sweden; 3 Department of Psychiatry and Psychotherapy, Campus Charité Mitte, Charité Universitätsmedizin Berlin, Berlin, Germany; 4 Department of Chemoinformatics, NovaMechanics MIKE, Piraeus, Greece; 5 Department of Medical Genetics, Oslo University Hospital, Oslo, Norway; 6 Centre for Precision Psychiatry, Division of Mental Health and Addiction, University of Oslo, and Oslo University Hospital Oslo, Oslo, Norway; 7 Global Alliance of Mental Illness Advocacy Networks Europe, Brussels, Belgium; 8 Department of Child and Adolescent Psychiatry, Institute of Psychiatry and Mental Health, Hospital General Universitario Gregorio Marañón, IiSGM, CIBERSAM, ISCIII, School of Medicine, Universidad Complutense, Madrid, Spain; 9 Department of Clinical and Experimental Sciences, University of Brescia, Brescia, Italy; 10 KU Leuven and University Psychiatric Center KU Leuven, Leuven, Belgium; 11 Department of Mental and Physical Health and Preventive Medicine, University of Campania Luigi Vanvitelli, Naples, Italy; 12 Universitat Pompeu Fabra, Barcelona, Spain; 13 Discipline of Psychiatry, Adelaide Medical School, University of Adelaide, Adelaide, Australia; 14 Centre for Genomic Regulation, The Barcelona Institute of Science and Technology, Barcelona, Spain; 15 Department of Psychiatry and Psychotherapy, Ev. Hospital Bethel, Bielefeld University, Bielefeld, Germany; 16 Mental mHealth Lab, Institute of Sports and Sports Science, Karlsruhe Institute of Technology, Karlsruhe, Germany; 17 Department of Medicine and Surgery, Kore University of Enna, Enna, Italy; 18 Department of Psychiatry, Paris Brain Institute - Institut du Cerveau (ICM), UMR 7225/UMRS 1127, Sorbonne University/CNRS/INSERM, DMU Neurosciences, Pitié-Salpétrièren, APHP, Paris, France; 19 Psychiatric Center Copenhagen, Copenhagen, and Department of Clinical Medicine, University of Copenhagen, Copenhagen, Denmark; 20 Department of Bioinformatics, Fraunhofer Institute for Algorithms and Scientific Computing, Sankt Augustin, Germany; 21 Department of Psychiatry, Psychotherapy, Psychosomatics and Medical Psychology, Division of Psychiatry I, Medical University of Innsbruck, Innsbruck, Austria; 22 Department of Psychosis Studies, King’s College London, London, UK; 23 Department of Psychiatry and Psychology, Institute of Neuroscience (UBNeuro), Hospital Clinic, University of Barcelona, IDIBAPS, CIBERSAM, Barcelona, Spain; 24 Fliedner Klinik Berlin, Berlin, Germany; 25 Institute of Neuroscience and Physiology, The Sahlgrenska Academy, University of Gothenburg, Gothenburg, Sweden; 26 Drora and Pinchas Zachai Division of Psychiatry, Sheba Medical Center, Ramat-Gan, Israel; 27 Department of Psychiatry and Psychotherapy, University Medical Center, University of Mainz, Mainz, Germany; 28 Department of Psychiatry, UMC Utrecht Brain Center, University Medical Center Utrecht, Utrecht, The Netherlands; 29 Department of Biomedical Sciences, Section of Neuroscience and Clinical Pharmacology, University of Cagliari, Cagliari, Italy; 30 Statistical Genetics, Max Planck Institute of Psychiatry, Munich, Germany; 31 Division of Affinity Proteomics, Department of Protein Science, SciLifeLab, KTH Royal Institute of Technology, Stockholm, Sweden; 32 Centre for Neuropsychiatric Genetics and Genomics, Cardiff University, Cardiff, UK; 33 Institut du Cerveau - Paris Brain Institute - ICM, Inserm, CNRS, APHP, Hôpital de La Pitié Salpêtrière, Sorbonne Université, Paris, France; 34 Department of Psychiatry, Psychosomatic Medicine and Psychotherapy, University Hospital Frankfurt am Main – Goethe University, Frankfurt am Main, Germany; 35 Department of Neuroscience, University of Turin, Turin, Italy; 36 Institute of Psychiatric Phenomics and Genomics (IPPG), University Hospital Munich, Munich, Germany; 37 Kairos GmbH, Bochum, Germany; 38 Department of Psychiatry, Psychosomatic and Psychotherapy, Brandenburg Medical School, Neuruppin, Germany; 39 Department of Psychiatry, University of Melbourne, Melbourne, Australia; 40 Department of Psychiatry, The Florey Institute of Neuroscience and Mental Health, Melbourne, Australia; 41 University Clinic of Psychiatry and Psychotherapy, University of Bern, Bern, Switzerland; 42 Bonn-Aachen International Center for IT (B-It), University of Bonn, Bonn, Germany; 43 Department of Mental Health and Addiction Services, ASST Spedali Civili of Brescia, Brescia, Italy; 44 Department of Molecular and Translational Medicine, University of Brescia, Brescia, Italy; 45 Genetics Unit, IRCCS Istituto Centro San Giovanni di Dio Fatebenefratelli, Brescia, Italy; 46 Department of Biomedical and Neuromotor Sciences, University of Bologna, Bologna, Italy; 47 Department of Psychiatry and Psychotherapy, Medical Faculty Mannheim, Central Institute of Mental Health, University of Heidelberg, Mannheim, Germany; 48 Department of Psychiatry, Amsterdam University Medical Center, Amsterdam, The Netherlands; 49 World Psychiatric Association, Geneva University Psychiatric Hospital, Geneva, Suisse; 50 Bipolar and Depressive Disorders Unit, Institute of Neurosciences, Hospital Clinic de Barcelona, University of Barcelona, IDIBAPS, CIBERSAM, Barcelona, Catalonia Spain; 51 Unit of Psychiatry, Department of Medical Sciences and Public Health, University of Cagliari, Cagliari, Italy; 52 Unit of Clinical Psychiatry, University Hospital Agency of Cagliari, Cagliari, Italy; 53 Department of Pharmacology, Dalhousie University, Halifax, Canada; 54 Hospital del Mar Research Institute, Barcelona, Spain; 55 Gender in Medicine, Institute of Sexology and Sexual Medicine, Department of Psychiatry and Psychotherapy, Campus Charité Mitte, Charité - Universitätsmedizin Berlin, Berlin, Germany; 56 Division of Mental Health, Northern Adelaide Local Health Network, SA Health, Adelaide, Australia; 57 Headspace Adelaide Early Psychosis, Sonder, Adelaide, Australia; 58 GGZ inGeest Mental Health Care, Amsterdam, The Netherlands; 59 Department of Psychiatry and Neuropsychology, School for Mental Health and Neuroscience, Maastricht University Medical Center, Maastricht, The Netherlands; 60 Department of Psychiatry, Faculty of Medical and Health Sciences, Tel Aviv University, Tel Aviv, Israel

**Keywords:** Treatment resistance, Depression schizophrenia, Early detection, Early treatment, Precision psychiatry

## Abstract

Schizophrenia (SCZ), bipolar (BD) and major depression disorder (MDD) are severe psychiatric disorders that are challenging to treat, often leading to treatment resistance (TR). It is crucial to develop effective methods to identify and treat patients at risk of TR at an early stage in a personalized manner, considering their biological basis, their clinical and psychosocial characteristics. Effective translation of theoretical knowledge into clinical practice is essential for achieving this goal. The Psych-STRATA consortium addresses this research gap through a seven-step approach. First, transdiagnostic biosignatures of SCZ, BD and MDD are identified by GWAS and multi-modal omics signatures associated with treatment outcome and TR (steps 1 and 2). In a next step (step 3), a randomized controlled intervention study is conducted to test the efficacy and safety of an early intensified pharmacological treatment. Following this RCT, a combined clinical and omics-based algorithm will be developed to estimate the risk for TR. This algorithm-based tool will be designed for early detection and management of TR (step 4). This algorithm will then be implemented into a framework of shared treatment decision-making with a novel mental health board (step 5). The final focus of the project is based on patient empowerment, dissemination and education (step 6) as well as the development of a software for fast, effective and individualized treatment decisions (step 7). The project has the potential to change the current trial and error treatment approach towards an evidence-based individualized treatment setting that takes TR risk into account at an early stage.

## Introduction

A key problem in mental health is that a significant proportion of patients suffering from major mental disorders shows resistance against drug therapy, affecting on average every third patient [1]. At present, patients showing early signs of treatment resistance do not receive adequate early intensive pharmacological treatment but instead undergo a stepwise trial-and-error approach to first, second and third-line treatments [2, 3]. This roots in three major problems in research and clinical translation: first, we lack effective methods to identify individuals at risk for treatment resistance (TR) early in the course of illness; second, we lack effective, personalized treatment strategies grounded in insights into the biological basis of TR and third, we lack efficient processes to translate scientific insights about TR into clinical practice and primary care. There are numerous attempts to define treatment resistance. In the best known and most established version for the field of depression, treatment resistance is defined as the failure of two or more pharmacological treatment attempts of sufficient duration and dosage[4]. This definition is used in particular by the European Medicines Agency and the US Food and Drug Administration. This is a pragmatic and clinical attempt at a definition. The Psych-Strata consortium is working on this complex topic and will also strive for research efforts with regard to a new definition of treatment resistance. However, the focus will be on a pragmatic and clinical definition.

**Fig. 1:**
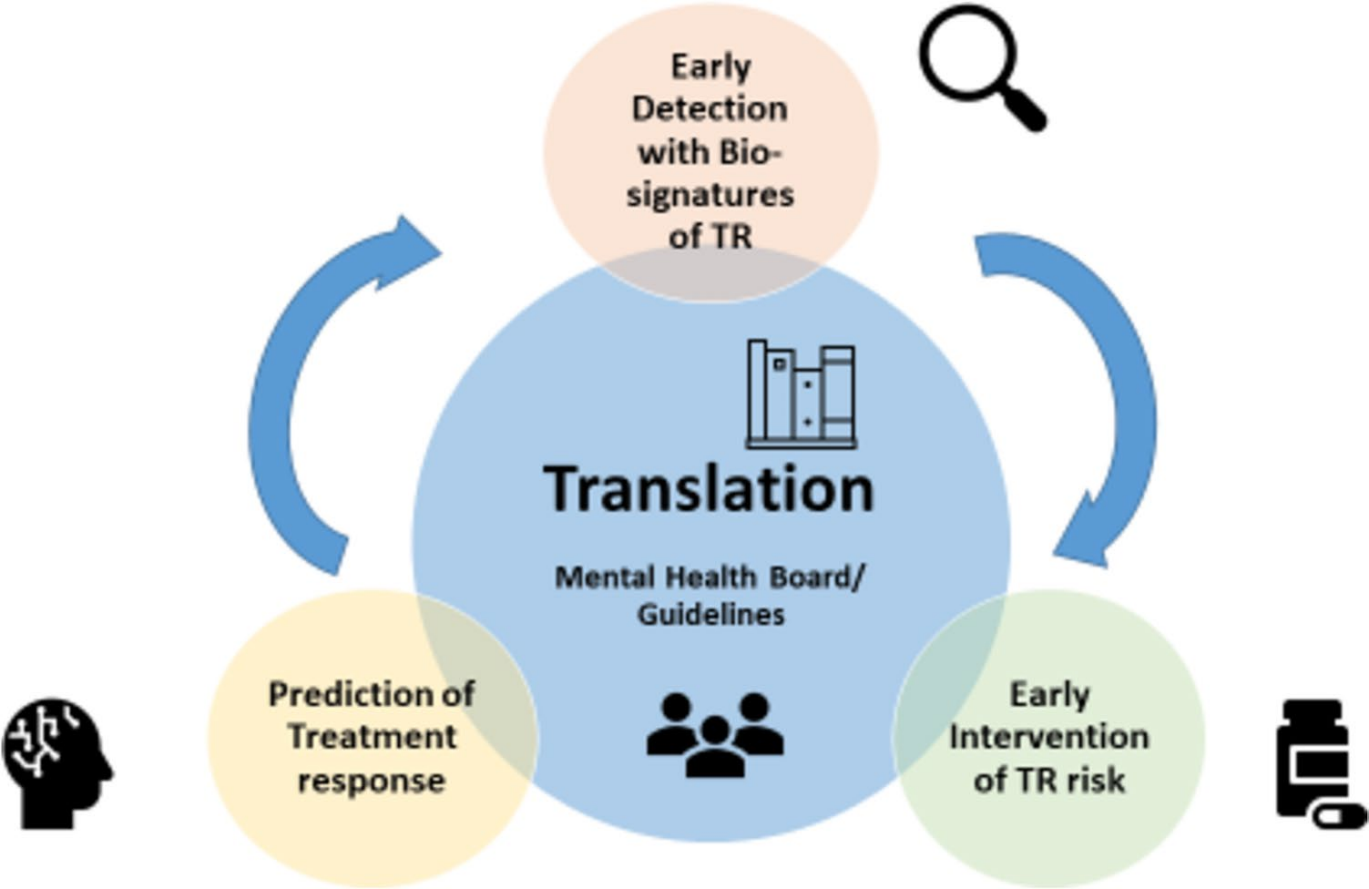
Outline of the Psych-STRATA overall concept

The Psych-STRATA consortium aims to address these three major problems and enable evidence-based identification and treatment of individuals at risk for TR (Fig. 1) across the three major psychiatric disorders, schizophrenia (SCZ), bipolar depression (BD) and major depressive disorder (MDD). To this end, the consortium will establish a comprehensive collection of biological, digital, cognitive and clinical data, including therapy-response and -outcome measures across SCZ, BD and MDD. Subsequently, the consortium will leverage this data resource to derive multi-layered, transdiagnostic biosignatures associated with TR. These signatures will be used to build multi-modal machine learning or artificial intelligence (ML/AI) models to predict risk of TR and ultimately allocate individual patients to optimal intervention strategies (Fig. 1). We are investigating the underlying genetic disposition to TR, which forms the basis of the interrelated and genetically driven transcriptomic and proteomic findings. Finally, these concepts will converge into interdisciplinary and patient-oriented decision-making mental health boards. Using a modern participatory approach, we aim to transfer our results into evidence-based clinical guidelines and better information for patients at risk.

### Overall Objectives

First, we will identify genetically-based biosignatures underlying liability to TR in SCZ, BD, MDD as well as transdiagnostically (objective 1). These genetic signatures will be complemented by novel multi-omic biomarkers, biosignatures, and clinical phenotypes underlying treatment outcome based on multi-omic profiling of longitudinal intervention cohorts (objective 2). Jointly, the deliverables of these two objectives will enable a multi-modal machine-learning model to predict TR risk and treatment outcome (objective 2).

Next, we will compare the efficacy and safety of early intensive pharmacological treatments to treatment as usual in SCZ, BD, and MDD patients in patients at risk for TR, initially defined as first-line treatment failure (objective 3), in a randomized controlled trial (RCT). The RCT will be accompanied by in-depth phenotyping during the treatment process. The retrospective analysis will then enable the validation and extension of the machine-learning model to predict risk for TR and treatment outcome (objective 4a). Moreover, detailed analysis will permit the identification of novel markers of treatment response as well as the validation of the polygenic risk score (PRS), and the multi-omic predictors in the RCT setting. Jointly, these efforts will give rise to a model to predict the probability of treatment response to early intensive pharmacological treatment after a first-line treatment failure (objective 4b).

**Fig. 2:**
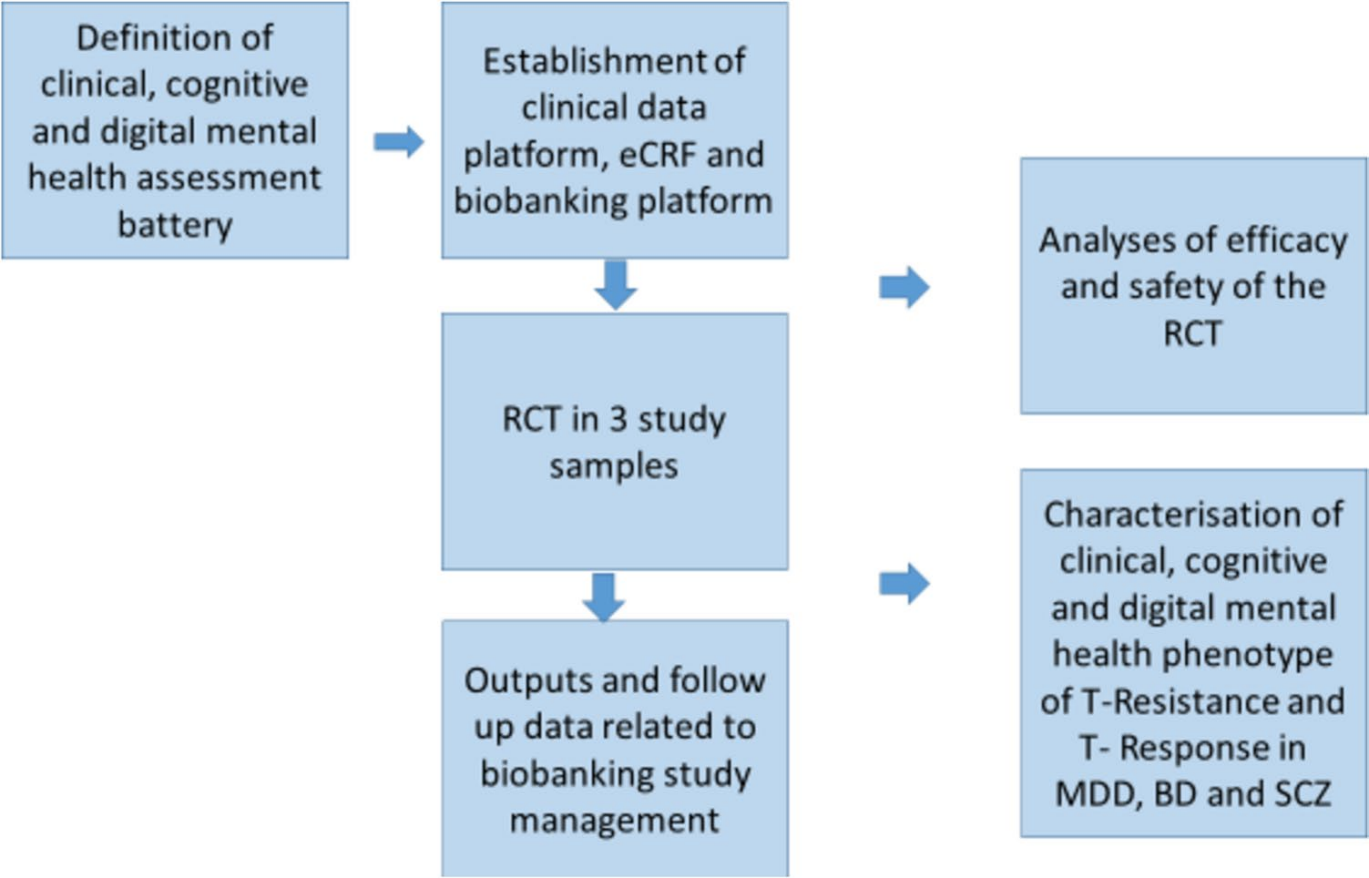
Overview of the main objectives of Psych-STRATA

Finally, we will develop and implement a novel framework of decision-making incorporating a multi-modal prediction tool and a shared decision-making process between mental health professionals, scientists, and patients, embedded in the context of a mental health board (objective 5a). In parallel, we will translate the results into guidelines for treatment (objective 5b) and disseminate them (objective 6). Figure 2 gives an overview about the main objectives and how they relate to each other. In the following, we present the seven steps in which we plan to pursue our objectives in more detail.

## Psych-STRATA in seven steps

### Step 1: identification of transdiagnostic genetic biosignatures underlying treatment resistance in SCZ, BD and MDD

We will derive genetically based biosignatures (e.g., PRS) associated with transdiagnostic TR in SCZ, BD, and MDD. This objective will also address the clinical need to identify the common biological basis of TR across these disorders and highlight genetics-based predictors for early identification of TR. These constitute essential components of any future clinical decision support system by providing genetic biomarkers for the early detection of individuals at risk for TR, eventually contributing to a substantial reduction in the trial-and-error period of medication evaluation in each patient.

We will utilize standard, established genome-wide association study (GWAS) and polygenic methods as well as exploiting new approaches in large, harmonized data sets; such as pharmacogenomic allele calling from array data using PyPGx [5]. To this end, the Psych-STRATA consortium brings together the largest collection of treatment resistant individual level genetic data to date, amounting to more than 150,000 cases (90,000 from biobanks).

We will perform within disorder GWAS of TR and refine optimal definitions of TR according to single nucleotide polymorphisms (SNP)-based heritability estimates, genetic correlation of definitions within and across disorders as well as polygenic predictive ability into the independent deeply phenotyped cohorts in step 2. In addition, we will perform cross-disorder GWAS for TR across SCZ, BD, and MDD using standard and cutting-edge methods [6] and will ascertain whether SCZ, BD, and MDD TR risk loci are disorder-specific or act across disorders (TR pleiotropic loci).

We will gain insights into the shared biology of TR by applying statistical fine-mapping to the TR GWAS results as well as downstream analyses at a single cell, tissue, actgene, and pathway level. These models will be used to pinpoint genes, pathways, cell types, and tissues associated with TR using transcriptome and pathway wide association analysis [7]. Methodically we will identify specific genes and biological pathways relevant to TR across disorders through gene prioritization methods, which combine state-of-the-art statistical fine-mapping [8] and co-localization of expression quantitative loci (eQTLs) [8], gene-set enrichment analysis framework [9] as well as functional mapping of with gene-set analysis (GSA)-MiXeR [10] within and across disorders.

In this step, we will also perform PRS analysis [11, 12] to assess liability to TR across all cohorts for (1) within-disorder TR (case/case) for SCZ, BD, MDD and (2) combined TR (case/cases) across diagnoses. We will leverage emerging methods (e.g., polygenic risk score–continuous shrinkage (PRS-CS), linkage disequilibrium (LDPRED), PRS of CHD-associated biomarkers (BioPRS)) [13] to identify the most powerful approach for assigning a genetic based biosignature risk score for TR across disorders and additionally consider the potential role of pharmacogenomic markers in mediating or moderating polygenic effects [14, 15] along with polygenic overlap with metabolic dysfunction [16]. We expect that PRS calculations will explain rather small but nevertheless significant proportions of the cohort variance. We will perform transcription (TWAS) and pathway activity level (PALAS) genome wide association studies for TR liability within and across disorders and leverage the results to compute tissue specific and multi-tissue gene and pathway level risk scores for each individual [12]. Additionally, recently reported analysis on polygenic liability for antipsychotic dosage and polypharmacy will be implemented [17].

**Fig. 3:**
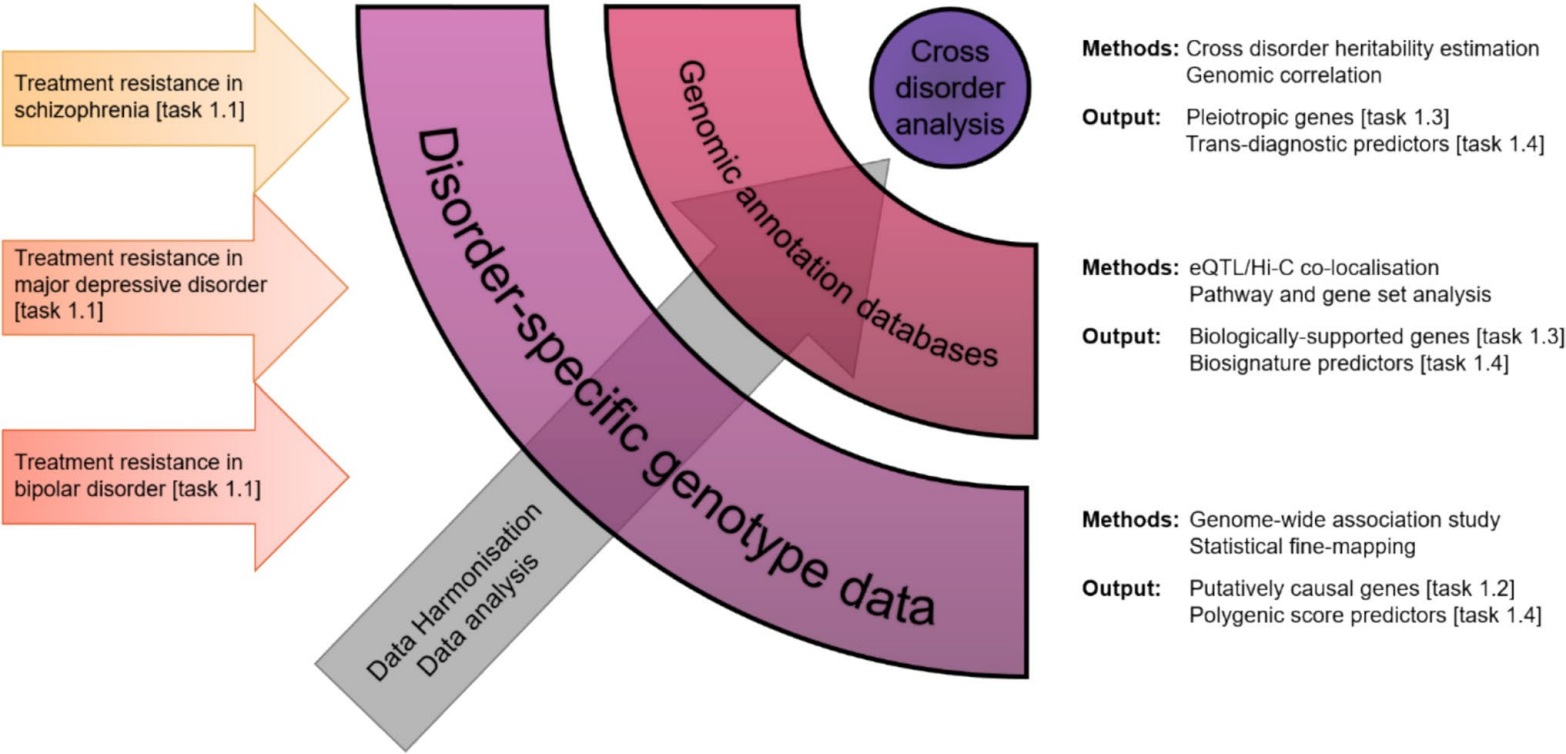
Overview of the analysis strategy of step 1

Finally, we will evaluate distinct biosignature scores (PRS, TWAS-PRS, etc.) with respect to their discriminatory power of TR vs non-TR patients individually as well as in combination. Figure 3 gives an overview about the step 1 analysis strategy.

### Step 2: identification of multi-modal biosignatures profiles associated with treatment outcome

In this step, we will first derive multi-omics biosignatures and biomarkers associated with treatment outcome and TR. We will conduct proteomic analyses on longitudinal cohorts of SCZ, BD, and MDD patients to assess treatment effects on the serum proteome. Using a multi-stage approach, we will examine over 5400 proteins in ~ 900 serum samples from TR/non-TR individuals during the discovery phase, leveraging the Olink Explorer HT panel [18]. This will elucidate proteomic changes linked to different treatment responses. Subsequently, we will establish a thoroughly selected candidate protein panel of up to 380 proteins for the validation phase. We plan to use the commercially available kits with 5000 proteins. This will consist of a high-throughput antibody-based suspension bead-array assay utilizing antibodies from the Human Protein Atlas, across approximately 6000 samples of TR/non-TR individuals with SCZ, BD, and MDD.

In the described datasets, we will aim to identify proteomic biomarkers associated with (1) response to specific treatments, depending also on sample size considerations, (2) response irrespective of treatment type, and (3) TR vs response for all patients and those with two measurement time points [19]. We will consider the use of both linear and non-linear methods for detecting possible associations and the use of strategies to limit multiple-testing burden, and validate the results of genetic analyses (Step 1). In detail, we will consider the following: (1) genes with evidence for association with TR based on TWAS analysis, (2) membership or interaction with TR associated pathways, (3) classification as druggable gene (e.g., Drugbank, https://go.drugbank.com/), and (4) proteomics-level pathway analysis. With the aim to integrate the different layers of data, we will also integrate genetic and proteomic data to identify protein quantitative trait loci (pQTL) associated with treatment outcomes.

These insights will then be combined with clinical and psychosocial variables to develop multi-modal predictors that integrate the different key components contributing to treatment outcomes. For this scope, we will evaluate different machine learning and integration strategies (e.g., multimodal neural networks) [20] to integrate *omics and clinical predictors (e.g., socio-demographics, severity and cause of illness, comorbidities, psychosocial function, clinical digital markers on sleep and activity).

Finally, we will leverage personalized disease models based on induced pluripotent stem cells (iPSCs) from TR/non-TR patients to validate the identified biomarkers/biosignatures [21], identify new biomarkers, and dissect the neurobiological mechanisms contributing to treatment outcomes. RNA-Seq and metabolome profiling of iPSC under baseline and exposure to first line drug treatments considered in Step 3 will be assessed. Finally, these results will be integrated with omics results of the previous steps, to pinpoint mutually validate the TR associated molecular changes/biosignatures.

### Step 3: randomized controlled trials on efficacy and safety of early intensified pharmacological treatment

**Fig. 4:**
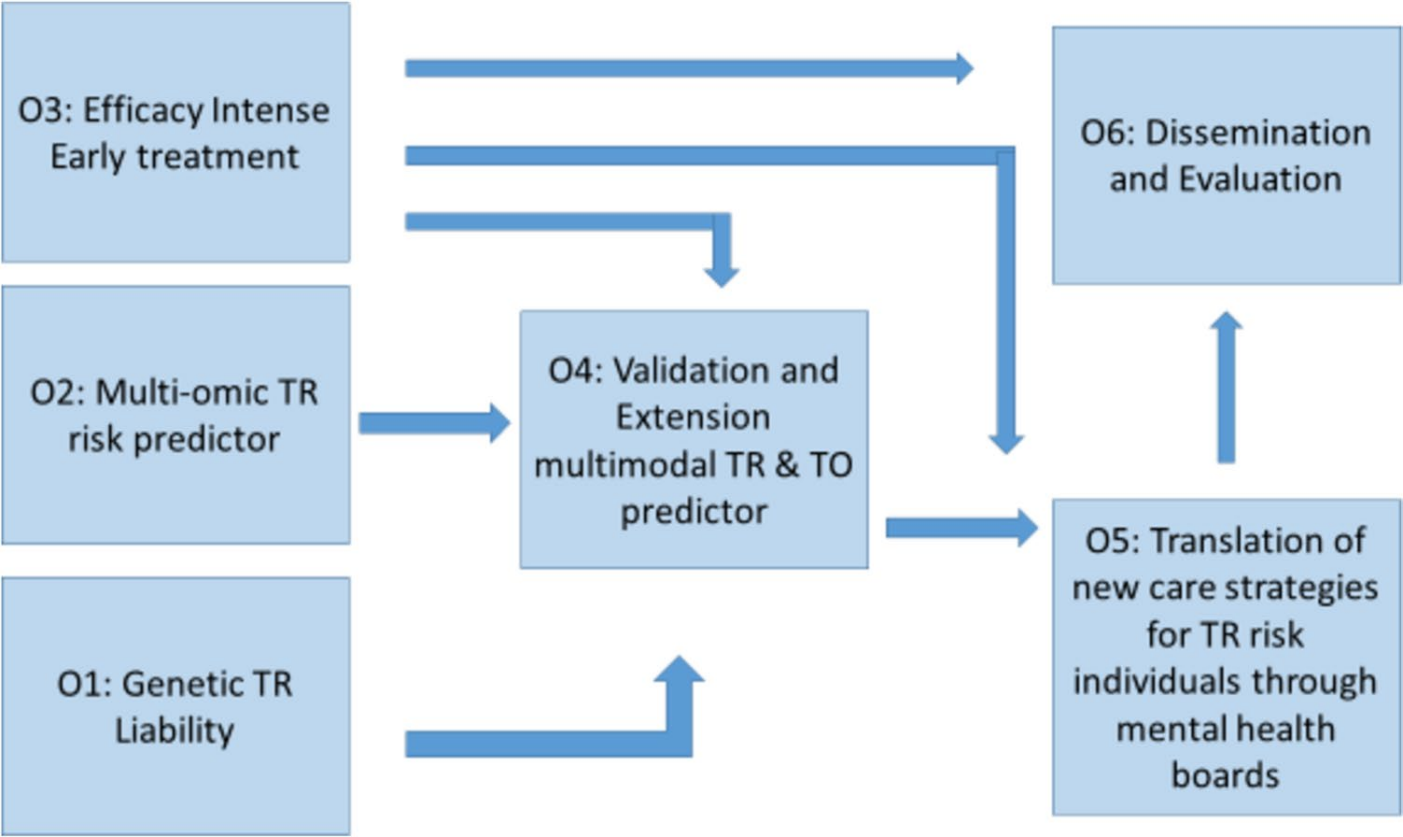
Overview of the randomized controlled treatment (RCT) strategy

The main objective of this step will be to conduct an innovative RCT for MDD, SCZ, and BD patients (strategy in Fig. 4). The trial will be conducted as an open-label study, with the assessors of the primary outcome measures blinded to treatment. Patients, whose illness did not respond to a first-line therapy in the current disease episode, will be randomized (1:1 randomization) to early intensified pharmacological treatment (EIPT) or treatment as usual (TAU). The treatment phase lasts 6 weeks, but it is an intent-to-treat approach, which means that patients might stop treatment earlier or could be treated longer. Follow-up is carried out up to 6 weeks after the end of the treatment phase.

EIPT will consist of a pharmacological treatment that is currently recommended for patients with TR by treatment guidelines (i.e., patients who did not sufficiently respond to ≥ 2 treatments). In MDD, a switch to another oral antidepressant plus esketamine (EIPT) will be compared with a switch to a second line antidepressant (TAU). In SCZ, a switch to clozapine (EIPT) will be compared with a switch to a second-line antipsychotic (TAU). For BD, a switch to one of the following will be compared: 1. escitalopram, sertraline, or venlafaxine plus 2. two of the following: lithium, lamotrigine, valproate, or quetiapine (EIPT) versus quetiapine plus lithium or valproate, or lamotrigine (TAU).

Treatment outcomes will be measured over a period of six weeks. The primary endpoint is the efficacy so called change in symptom severity (according to standard scales); the secondary endpoints are cognition and multiple patient-centered outcomes such as quality of life, general functioning, and life satisfaction. Side effect profiles and long-term effects will also be assessed until week 12 after treatment. The total follow-up period will be three months, comprising five clinical study appointments; the baseline visit will follow within a week after the screening, and visits 3, 4 and 5 will be conducted at 2 weeks, 6 weeks, and 12 weeks post-baseline, respectively.

In addition to a comprehensive medical history, and numerous clinical self-assessment, and clinician assessment instruments, there are three special features of this RCT, as we will include a comprehensive assessment of cognition, digital markers, and biomarkers (blood and feces). Genomic and proteomic analyses will be performed for integration/validation of steps 1 and 2 (see the next paragraph). The digital biomarkers will be collected using electronic diaries, mobile sensor technology, and wearables, in some cases via smartphone.

The efficacy of the medication will be measured through the Positive and Negative Symptom Scale (PANSS, SCZ) and Montgomery Åsberg Depression Rating Scale (MADRS, MDD, and BD) and compared between both arms of the trial. The safety will be measured through General Assessment of Side Effects (GASE). A further aim of this step is to identify clinical and digital psychological predictors of treatment response/TR. Other key data of interest are cognitive phenotypes potentially associated with treatment response, and the digital mental health data will be analyzed to identify digital measures associated with treatment outcomes [22, 23].

### Step 4: identification of multi-modal signatures associated with TAU and early intensified treatment response for evidence-based treatment recommendations in clinical practice

This step will integrate and extend the results of step 1–step 3. First, we will leverage the multi-modal data from the RCT in step 3 to validate identified genes, pathways, proteins, pharmacogenomic alleles, and genetic scores predictive of treatment outcome based on steps 1 and 2. More specifically, we will validate biosignature based biomarkers (genetic and proteomic) associated with TR liability and treatment outcome in SCZ, BD, and MDD. We will compute PRS and genetic/multi-omic based biosignatures defined in step 1 and 2, respectively, for all RCT patients at baseline. We will also perform multi-omic integration to validate biomarker candidates from joint step 1, step 2, and RCT cohorts across molecular layers and explore the predictive capacity of multi-omic features with respect to treatment outcome.

Second, we will conduct a further biomarker discovery effort based on the data collected in step 3 to identify transcriptomic or proteomic biomarkers based on their capacity to classify SCZ, BD, and MDD patients into responder/non-responder groups. We will target genes and serum proteins that are associated with 1. response to TAU, 2. response to EIPT, and 3. biomarkers predicting differential response to TAU vs EIPT in each disorder and transdiagnostically. In a complementary effort, we will connect the distinct molecular layers and also identify expression and protein quantitative trait loci (eQTLs and pQTLs) under baseline and post-treatment conditions [24] to pinpoint eQTLs/pQTLs associated with treatment response.

Finally, we will build on the validated biosignatures from step 1 and 2 as well as the latter results and establish a multi-modal machine-learning model to predict treatment outcome for TAU or EIPT. To this end, we will extend the multi-model machine-learning model developed in step 2 and integrate genetic, transcriptomic, and proteomic profiling of steps 1–3 with deep clinical phenotyping data to build a multi-layered treatment response model for TAU/EIPT. More specifically, we will combine the following data modalities and evaluate their contribution to overall prediction performance:

- genetic based scores and/or pharmacogenomic alleles associated with TR found in step 1,
- omics based biomarker scores for TR found in step 2,
- omics based individual and aggregated biomarker scores of response to TAU vs EIPT,
- clinical, psychosocial, and neurobiological measures (symptom rating, cognition, etc.), and
- digital mental health features.

These data modalities will be integrated using different early (e.g., canonical correlation analysis, multi-modal autoencoders), intermediate (e.g., multi-modal neural networks) and late (e.g., stacking) integration strategies for multi-modal data. We will evaluate candidate features with respect to added value via nested-cross validation designs.

In addition, patient trajectory modeling and simulation will be implemented in this step, using e.g., the previously published Variational Autoencoder Modular Bayesian Networks (VAMBN) and/or MultiNODE algorithms [25, 26]. We will evaluate statistical dependencies between biomarkers, clinical outcomes, and digital assessments and generate virtual patients to simulate different treatment scenarios. Thus, this step will provide the data and analysis groundwork for the evidence-based treatment recommendation component of the mental-health board in clinical practice (step 5).

### Step 5: development of a treatment decision-making mental health board

In this step, we will focus on three main objectives. The first one is the implementation of a software tool embedding AI/ML models developed in step 4 for treatment decision support. This software will help clinicians to obtain predictions of treatment outcome for individual patient using their specific characteristics (multi-omic, clinical, and digital data). Model predictions will be explained via methods such as Shapley Additive Explanations (SHAP). Moreover, we will embed interactive features, e.g., mapping of omics-derived features to molecular networks. For example, these networks can include known drug-target interactions, retrieved from public databases (e.g. Drug Bank), hence pointing to potential alternative treatments. Finally, we will evaluate this software tool regarding its technical correctness and its clinical utility.

The second objective is the creation of a decision-making mental health board, which aim will be to conceptualize and facilitate a shared decision-making (SDM) process between patients, carers, clinicians, and scientists. We will realize this in three phases. The first will be the development of a shared decision-making platform, which we will evaluate by running focus groups and surveys. We will also analyze and evaluate the potential impact and obstacles of implementing biosignature-assisted SDM processes. Another important goal is the implementation of the decision-making mental health board. It is anticipated that this board will consist of an interdisciplinary group of scientists, clinicians, primary care physicians, psychotherapists, patients, and carers. We will conduct a pilot study utilizing the decision-making mental health board. The outcomes of this pilot study will provide first experience of the mental health board in national and international clinical settings.

The last objective of this step is the creation of a framework for implementation of treatment guidelines. For this, we aim to develop definitive, prospective level evidence [27]. We will convene an expert working group (clinicians, researchers, and patient representatives) to review the existing evidence [28, 29] and submit a consensus report to the major international guidelines. We will also prepare a publication.

### Step 6: patient empowerment, dissemination, and education

The last step of Psych-STRATA focuses on patient empowerment, dissemination, and education in different ways. First, we will generate opinions maps at the European level regarding the use of digital apps and continuous mental health assessments among relevant stakeholders. We will integrate app-based surveys in order to collect participant feedback from the RCT (step 3). Likewise, we will develop surveys on the same questions for other stakeholders: participants in other clinical trials, relatives, clinicians, researchers, policy makers, and any other relevant group of individuals.

Survey participants will be recruited via channels such as the GAMIAN-Europe’s website, touching base with local contacts of each patient group embedded in GAMIAN-Europe, presentations at local and international meetings, newsletters, and social media. Another important objective will be the comprehensive evaluation of the novel mental health board with shared decision-making (step 5), using a structured survey that will target relevant stakeholders.

The last objective is the dissemination of findings and education. The World Psychiatric Association (WPA) will be an important partner in the dissemination and education part (workshops, surveys, connecting with groups around the globe). Part of this is setting up and maintaining the official Psych-STRATA website (https://psych-strata.eu/), Psych-STRATA Twitter/X account (@psych_strata), and a LinkedIn account. We are currently creating and updating communication information material, developing project factsheets, posters, official presentations, and all types of dissemination materials including newsletter (https://psych-strata.eu/newsletter/). Moreover, we will organize workshops for healthcare providers and disseminate our expert consensus document. Lastly, press releases will be issued when significant results have been achieved to reach and inform specialized and/or generalist media on key activities and results.

### Step 7: in silico drug repurposing for identifying potential therapeutics in SCZ, MDD, and BD and development of the Psych-STRATA software as a service (SaaS) platform

This step represents a transformative approach to in silico drug repurposing, integrating advanced computational techniques such as cheminformatics, text mining, network pharmacology, omics data analysis, and network-based drug repurposing methods. By adopting these methods, Psych-STRATA aims to introduce a rational approach to drug identification, improving the precision in identifying potential drug candidates for conditions like SCZ, MDD, and BD [30]. Unlike traditional trial-and-error methods, computational simulations and cheminformatics tools enable the prediction of drug efficacy, bioavailability, and potential side effects before clinical trials, thereby streamlining the drug development process [31]. Moreover, network-based techniques consider the complex interplay of biological pathways involved in mental disorders [32], which is crucial for conditions like SCZ, MDD, and BD that often share common biological pathways. By mapping these pathways and identifying key nodes or targets, Psych-STRATA can suggest existing drugs effective across multiple conditions, aligning with the emerging paradigm of poly-pharmacology [33]. These methodologies enable the exploration of latent linkages between different mental disorders, facilitating drug repurposing by identifying drugs effective for conditions not initially targeted.

Moreover, the Psych-STRATA SaaS platform represents a significant methodological advancement, offering a dedicated platform for seamless integration of ML/AI into healthcare providers’ daily decision-making processes [34]. This platform aims to provide clinicians with more personalized, actionable data, enabling real-time, data-driven decision-making in mental health care, which is often lacking in current systems. By offering patient-specific insights, particularly crucial for complex mental health disorders like SCZ, MDD, and BD, the platform enhances the quality of care while reducing the burden on healthcare systems. The integration of advanced ML/AI models into clinical practice streamlines decision-making, making diagnosis and treatment planning quicker and more efficient. Ultimately, by offering personalized data and enabling real-time decision-making, the Psych-STRATA platform has the potential to revolutionize mental health care provision, setting new standards in the field.

## Discussion

The long-term vision of Psych-STRATA is to pave the way for a shift from the current state-of-the-art trial-and-error and stepwise treatment paradigms towards evidence-based treatments tailored and stratified according to the individual person with SCZ, BD, or MDD at risk for TR early in the treatment process, and thus preventing further complications such as chronicity and suicide risk [35].

Psych-STRATA will contribute to a better understanding of the genetics and molecular biological basis of TR and TR liability in individuals suffering from SCZ, BD, and MDD. In particular, this project will deliver (transdiagnostic) genes and pathways underlying TR as well as molecular genetics and environmental modulators of treatment outcome, combining molecular, clinical, and digital patient assessments.

Approaches that use artificial intelligence methods and focus on MRI data in combination with clinical data are also the subject of current research innovations. Here, too, promising results are emerging [36–38], for example in affective disorders and schizophrenia. However, these approaches are not yet ready to be used in clinical practice [36, 39], as the necessary validation and assessment of clinical benefit have not yet been achieved [36]. Blood-based approaches for predicting treatment success have the advantage that they are cheaper, faster and more economical than MRI-based methods and therefore fulfill important criteria for promising biomarkers. In our view, blood-based approaches are an important complement to imaging-based approaches. Both are promising biomarkers which, especially in combination, may also be indicative for personalized psychiatry. For this reason, we will also endeavor to establish and intensify collaborations with neuroimaging research as part of the project.

The project will provide researchers and mental health professionals with the ability to detect SCZ, BD, and MDD patients at risk for TR early in an evidence-based fashion beyond mere symptom-based criteria. This process will be based on novel individual biomarkers and most importantly, on multi-modal predictive models of TR risk and treatment response by integrating clinical, digital, and multi-omics information as individual biomarkers are expected to have limited predictive power [40]. The project will empower professionals to treat at-risk patients early and more accurately, minimizing the time that treated individuals spend taking ineffective medications or at high risk of adverse drug reactions. The establishment of a framework and solid database as well as a mental health decision board will enable a patient-oriented approach, the translation of early detection of TR and tailored treatment concepts from primary care towards specialist care.

The project findings can be directly incorporated into the development of new evidence-based guidelines for the pharmacological treatment of patients after an initial treatment failure, which can be an important contribution to reduce the transition to TR and the chronicity of mental illness. The project has a good chance to change the evidence base for future treatment guidelines for TR, to reduce suffering for individuals and societal burden in the long run.
